# Food classifications provide an approximate packaging indicator to support monitoring of mismanaged plastic waste

**DOI:** 10.1038/s41598-025-89350-0

**Published:** 2025-02-11

**Authors:** Mawuli Dzodzomenyo, Moses Asamoah, Joseph Okotto-Okotto, Lorna-Grace Okotto, Peggy Wanza, Gustavus A. Myers-Hansen, Jim Wright

**Affiliations:** 1 School of Public Health, University of Ghana, Accra, Ghana; 2 Social Statistics and Demography, University of Southampton, Southampton, UK; 3 Victoria Institute for Research on Environment and Development International, Rabuor, Kenya; 4 School of Spatial Planning and Natural Resource Management, Jaramogi Oginga Odinga University of Science and Technology, Bondo, Kenya; 5 School of Geography and Environmental Science, University of Southampton, Southampton, UK

**Keywords:** Food groups, Household expenditure surveys, Plastic waste, Sustainable cities and communities, Environmental impact, Sustainability, Environmental impact

## Abstract

Mismanaged plastic waste undermines environmental quality, aquatic ecosystems and ultimately public health. Mismanaged plastics increasingly originate from urban populations lacking waste collection services. Household expenditure survey analyses have quantified plastic waste generation among these populations, but only for individual products (e.g. bagged or bottled drinking-water), not for the overall packaging profile of households’ basket-of-goods. This study aims to evaluate how far the international NOVA classification of foods/beverages and commodity classifications by residents predict plastic food packaging. Via a cross-sectional market surveillance survey, packaging was observed for 502 and 396 transactions at selected retail outlets serving low-income areas of Greater Accra, Ghana, and Kisumu, Kenya respectively. In both cities, NOVA processed/ultra-processed food and food/beverage classes created by local residents were significantly associated with greater plastic food packaging, with similar predictive performance. Plastic packaging prevalence was also significantly higher in supermarkets. Plastic packaging use was significantly lower in Kisumu, where single-use carrier bags are banned. Thus, given their international availability and national representativeness, household expenditure surveys have potential for monitoring to inform policy by targeting plastic pollution hot-spots. However, survey-based monitoring should incorporate expert knowledge of national context and the retail environment to reflect the realities of packaging use.

## Introduction

Target 11.6 of Sustainable Development Goal (SDG) 11 seeks to reduce by 2030 “the per capita environmental impact of cities…. including by paying special attention to air quality and municipal and other waste management”^1^. Plastic waste management is of particular concern: 275 million tons of plastic waste were generated globally in 2010, of which between 4.8 and 12.7 million tons entered the oceans^2^. Some forecasts predict that this could increase by up to 90 million tons by 2030^3^. Mismanaged plastic waste not only causes localised harm such as blocking urban storm-drains and posing a hazard to wildlife, but as plastic debris deteriorates into micro-plastics, these can bio-accumulate in marine organisms^4^ and ultimately in human tissue such as breast-milk and placentas^5^. Low and middle-income country (LMIC) cities, where waste service coverage is low^6^ but life-styles are changing, are increasingly a source of mismanaged plastics. Given this context, the United Nations Environment Programme has developed a plastic pollution hot-spotting workflow, which can be used to inform action^7^. This approach systematically identifies the economic sectors, locations, polymers, applications or products, and waste management processes that most contribute to plastics leaking into the environment.

To support plastic pollution hot-spotting, household expenditure surveys can provide insights into mismanaged waste from individual plastic products by combining details of plastic product consumption with information about household solid waste management^8^. Several hundred such household surveys have been implemented worldwide that quantify expenditure, including on foods and beverages. The most widely implemented are Household Budget Surveys (HBS), Income and Expenditure Surveys (IES), and the World Bank’s Living Standards Measurement Surveys (LSMS)^9^. Their analysis enables consumption monitoring of products in plastic packaging among populations lacking solid waste disposal services and thereby plastic waste hot-spot identification. Nationally and regionally representative waste generation totals can be estimated from consumption of specific products among populations lacking solid waste management services. Studies have quantified plastic waste from packaged drinking-water consumption^10^ and disposable diaper waste^11^ in such populations, using these as case study products. Additionally, where household expenditure surveys are georeferenced, it is possible to map geographic hotspots where plastic waste from a particular product is mismanaged^12,13^.

To date, however, individual products containing plastics have been identified on a somewhat ad hoc basis based on the availability of suitable household survey data and nature of the product^8^. Thus, analyses have focused on individual products sold in standardised plastic packaging, rather than considering the packaging waste generated by the full basket-of-goods that a household consumes. For example, in Ghana and Nigeria, 500mL of water is sold packaged in a plastic sleeve, comprising 2g of high or low density polyethylene (HDPE or LDPE) plastic, enabling estimation of primary plastic packaging used by households lacking solid waste disposal services^10^. Given that multinational companies now explicitly target LMIC markets by using sachets as more lightweight, affordable packaging than polyethylene terephthalate (PET) bottles^14^, rising water sachet consumption in West Africa may exemplify a broader, worldwide packaging trend. In contrast, analyses of other products such as cooking oils^8^ suggest that their packaging varies greatly, sometimes being pre-packaged but also sold by informal traders in reused containers, for example by refilling beverage bottles with oil. Analyses of household expenditure surveys for other purposes classify the entire household expenditure profile, aggregating across all food items. For example, nutritionists have identified the number of food groups that households report purchasing to assess dietary diversity^15^. More recent studies^16,17^ have applied the NOVA food classification to food and beverage purchases reported via household expenditure surveys. Given a global dietary shift towards processed foods with longer shelf-life, this classification differentiates unprocessed or minimally processed foods, processed culinary ingredients, and processed foods from ultra-processed foods and drinks^18^. However, these studies have only considered the nutritional but not waste management impacts of processed food consumption, which arise from their plastic packaging.

In principle, it may thus be possible to systematically characterise the plastic packaging associated with an entire basket-of-goods reported via a household expenditure survey, classifying commodities purchased to create a plastic waste generation index. Since many countries have conducted multiple, successive household expenditure surveys, such an index could potentially be used to monitor plastic waste generation trends. Where household surveys are georeferenced, an index could also potentially be used to map geographic hotspots of mismanaged plastic waste. Finally, such an index could enable more systematic identification of specific products for more detailed follow-up investigation to inform solid waste management, product redesign, and extended producer responsibility. Such investigations could target those products consistently sold in single-use plastics to households lacking solid waste disposal services. However, such an approach would require successful classification of survey commodity codes into groups that approximately reflect their packaging.

In this paper, we therefore use market surveillance fieldwork to identify products sold in plastic packaging, addressing the following research questions:

- How extensive is plastic packaging of foods and beverages in slum neighbourhoods of contrasting two LMIC cities across different types of retail outlet?
- To what extent does the NOVA classification predict packaging of foods or beverages in plastics?
- Can residents who are familiar with a city’s retail environment categorize foods into broad groups based on their likelihood of being packaged in plastics?

## Results

### Characteristics of retail outlets, products and their packaging observed through market surveillance

In Kisumu, the retail outlets surveyed comprised three supermarkets (2.9% of outlets), 37 open markets (35.4%), 17 kiosks (16.2%), and 48 shops (45.7%). In Greater Accra, where there was no direct equivalent to the Kenyan kiosk, outlets surveyed comprised 35 supermarkets (34.3%), 14 open markets (13.7%), and 53 shops (52.0%).

In Kisumu, market surveillance teams observed transactions involving 67 out of 191 detailed commodity codes (used in the 2015–16 Kenya Integrated Household Budget Survey, KIHBS), with some specific types of meat, fish and tuber not observed. In Greater Accra, market surveillance teams observed transactions involving 40 out of 72 coarse commodity codes (from the 2017 Ghana Living Standards Survey 7, GLSS7), with some specific types of meat, tuber and flour/grain not observed during market surveillance.

In Greater Accra, survey teams observed significantly more packaging by retailers than in Kisumu (Table 1). Consumers in Kisumu sometimes packaged their own purchases, but not in Greater Accra. The proportion of purchases of minimally or unprocessed food purchases (NOVA group 1) observed was similar in both cities, but more purchases of culinary ingredients (NOVA group 2) and fewer processed or ultra-processed food purchases (NOVA groups 3 and 4) were observed in Kisumu. Plastic packaging of foods or beverages was significantly more prevalent in Greater Accra (94.0%) than in Kisumu (56.7%), with more beverage purchases also observed.

**Table 1:** Characteristics of food/beverage transactions and packaging observed during market surveillance survey.

Foods/beverages	Kisumu: no plastic	Greater Accra: no plastic	*P* value (chi square test)
Sold without packaging	8 (2.1)	1 (0.2)	
Packaged by retailer when sold	67 (17.9)	157 (31.3)	
Pre-packaged	264 (70.6)	344 (68.5)	
Packaged by consumer	35 (9.4)	0 (0.0)	< 0.001
FAO GIFT Food groups			
Beverages	47 (12.6)	169 (33.7)	
Cereals & their products	79 (21.1)	79 (15.7)	
Milk/eggs & their products	51 (13.6)	33 (6.6)	
Fats & oils	38 (10.2)	37 (7.4)	
Fruits/vegetables/tubers & their products	28 (7.5)	47 (9.4)	
Fish/meat & their products	2 (0.5)	3 (0.6)	
Spices & condiments	34 (9.1)	29 (5.8)	
Sweets & sugars	84 (22.5)	96 (19.1)	
Pulses, seeds & nuts/savoury snacks	11 (2.9)	9 (1.8)	< 0.001
NOVA food groups			
1: Unprocessed or minimally processed	153 (40.9%)	214 (42.6%)	
2: Processed culinary ingredients	97 (25.9%)	79 (15.7%)	
3: Processed foods	1 (0.3%)	21 (4.2%)	
4: Ultra-processed foods	123 (32.9%)	188 (37.5%)	< 0.001
Main packaging material^1^			
None	43 (11.5)	1 (0.2)	
Metal	1 (0.3)	1 (0.2)	
Glass	12 (3.2)	3 (0.6)	0.005
Paper/cardboard^2^	107 (28.6)	22 (4.4)	< 0.001
Plastic	212 (56.7)	471 (94.0)	< 0.001
Not recorded	3 (0.8)	24 (4.8)	0.69
Small (Kadogo) transaction: quantity purchased in lowest quartile	37 (9.9)	52 (10.9)	0.64
Total	374	502	

^1^Percentages do not sum to 100 as multiple materials sometimes recorded for a given transaction; packaging material used by consumer not recorded below. ^2^ Includes beverage cartons comprising aluminium/card/plastic composites.

### Comparison of packaging classifications for foods and beverages

When packaging classifications of commodity types were compared (Table 2), there was moderate agreement (Kappa index of agreement between 0.41 and 0.60^19^) between the Kenyan resident’s classifications of broad, harmonised and detailed food/beverage codes. Similarly, there was moderate agreement between the Greater Accra resident’s packaging classification of food/beverages when sold via shops and open markets. However, there was no significant agreement between any of the food/beverage packaging classifications for Greater Accra versus Kisumu. Similarly, in Greater Accra, there was no agreement between the resident’s food/beverage packaging classification for supermarkets versus open markets or shops, with flours in particular classified differently.

**Table 2:** Agreement between residents’ classification of harmonised commodity codes versus detailed commodity codes for foods/beverages sold at open markets, supermarkets, and other retail outlets.

1st classification	2nd classification	No. commodity codes	% overall agreement	Kappa index of agreement (95% CI)	*P*-value (Kappa)
Kenya resident classification: KIHBS (detailed) codes	Kenya resident classification: harmonised codes	66	80.3	0.54 (0.34 to 0.74)	< 0.001
Greater Accra resident classification: supermarkets	Kenya resident classification: harmonised codes	65	63.1	0.05 (− 0.13 to 0.23)	0.31
Greater Accra resident classification: open markets	Kenya resident classification: harmonised codes	65	21.5	− 0.03 (− 0.13 to 0.07)	0.73
Greater Accra resident classification: shops	Kenya resident classification: harmonised codes	65	27.7	0 (− 0.11 to 0.11)	0.51
Greater Accra resident classification: supermarkets	Greater Accra resident classification: open markets	72	15.3	− 0.04 (− 0.11 to 0.03)	0.85
Greater Accra resident classification: supermarkets	Greater Accra resident classification: shops	72	26.4	0.05 (− 0.02 to 0.12)	0.10
Greater Accra resident classification: open markets	Greater Accra resident classification: shops	72	75.0	0.46 (0.30 to 0.62)	< 0.001

In Greater Accra, NOVA classes were significantly associated with resident-derived packaging classes for other retail outlets (Fisher’s exact test = 0.003, n = 72), but not for open markets or supermarkets (Fisher’s exact test = 0.049 and 0.459). At supermarkets, the local resident classified the majority of all NOVA food groups as being mostly packaged in plastic, whereas for open markets and other types of shop, the resident identified NOVA group 4 (ultra-processed foods) as having the most widespread use of plastic packaging. The Kisumu resident identified plastic packaging in all classes, but somewhat less in NOVA group 1b (minimally processed food), though the association was not statistically significant (Fisher’s exact test = 0.057; n = 65).

### Plastic packaging variation by product

Univariate logistic regression models for Kisumu (Table 3) showed significantly lower odds of commodities sold at kiosks being packaged in plastics. NOVA groups 2 (processed culinary ingredients) and groups 3 and 4 (processed or ultra-processed foods) had significantly higher odds of being packaged in plastics than minimally or unprocessed foods. As anticipated, the ‘sometimes’ and ‘seldom sold in plastic’ classes had lower odds of plastic packaging than the ‘mostly’ class, both for coarser harmonised commodity codes and for the more detailed codes from the KIHBS. ‘Kadogo’ transactions involving small food quantities had somewhat lower odds of plastic packaging, but this was not statistically significant. After adjusting for the effect of retail outlet type, all food classification and retail outlet effects remained significant. Results were very similar when considering manufacturers’ plastic pre-packaging only but excluding plastic packaging used by retailers (Supplementary Table S1). Overall goodness-of-fit was very similar for the adjusted logistic regression models based on classification by residents of coarse and detailed food codes and via NOVA (AIC = 1.25, 1.28, and 1.29 respectively; Supplementary Table S2).

**Table 3:** Odds ratios from unadjusted and adjusted logistic regression models, predicting plastic packaging of foods and beverages (by manufacturers or retailers) in Kisumu.

	Unadjusted		Adjusted	
Transaction characteristics	Odds ratio (95% CI)	*P* value	Odds ratio (95% CI)	*P* value
Small (‘kadogo’) transactions				
Quantity purchased below 25^th^ centile for commodity	0.61 (0.29–1.29)	0.20		
Facility type (reference: supermarket)				
Open market	1.08 (0.71–1.64)	0.73		
Kiosk	0.33 (0.19–0.60)	< 0.001		
NOVA food group (reference: Group 1 unprocessed foods)				
Group 2: Processed culinary ingredients	2.34 (1.38–3.99)	0.002	2.10 (1.22–3.64)	0.008
Group 3 or 4: processed or ultra-processed foods	4.34 (2.58–7.29)	< 0.001	3.93 (2.31–6.71)	< 0.001
Local resident classification: harmonised commodity codes (reference: mostly sold in plastics)				
Sometimes sold in plastics	0.42 (0.23–0.80)	0.008	0.39 (0.21–0.73)	0.003
Seldom sold in plastics	0.03 (0.00–0.18)	< 0.001	0.02 (0.00–0.15)	< 0.001
Local resident classification: KIHBS commodity codes (reference: mostly sold in plastics)				
Sometimes/seldom sold in plastics	0.34 (0.22–0.53)	< 0.001	0.30 (0.14–0.47)	< 0.001

Given the high prevalence of plastic packaging in Greater Accra, its logistic regression model coefficients and related odds ratios often had wide confidence intervals (Table 4). In univariate models, shops and open markets had significantly lower odds of selling foods/beverages packaged in plastics. NOVA groups 3 or 4 (processed or ultra-processed foods) had significantly higher odds of being packaged in plastics, an effect that remained significant in a model adjusted for retail outlet type. As anticipated, the resident-derived ‘sometimes sold in plastics’ class had significantly lower unadjusted odds of plastic packaging compared to the ‘mostly sold in plastics’ class. However, this effect was no longer significant in the adjusted model. The odds of ‘kadogo’ transactions involving small food or beverage quantities were not significantly different from larger bulk transactions. Model outputs were very similar when considering only pre-packaging by manufacturers, not plastic packaging by retailers (Supplementary Table S3). Overall goodness-of-fit was again similar for adjusted models based on the resident-derived versus NOVA classifications (AIC = 0.44 versus 0.42 respectively; Supplementary Table S2).

**Table 4:** Odds ratios from unadjusted and adjusted logistic regression models, predicting plastic packaging of foods and beverages (by manufacturers or retailers) in Greater Accra.

	Unadjusted		Adjusted	
Transaction characteristics	Odds ratio	*P* value	Odds ratio	*P* value
Small (‘kadogo’) transactions				
Quantity purchased below 25^th^ centile for commodity	3.33 (0.49–22.54)	0.217		
Facility type (reference: supermarket)				
Open market	0.16 (0.04–0.66)	0.011		
Shop	0.21 (0.06–0.70)	0.011		
NOVA food group (reference: Group 1 unprocessed foods)				
Group 2: Processed culinary ingredients	0.63 (0.26–1.55)	0.319	0.64 (0.24–1.67)	0.364
Group 3 or 4: processed or ultra-processed foods	9.46 (2.09–42.78)	0.004	9.20 (2.02–41.80)	0.004
Local resident classification (reference: mostly sold in plastics)				
Sometimes sold in plastics	0.29 (0.12–0.68)	0.004	0.49 (0.23–1.04)	0.066
Seldom sold in plastics	2.02 (0.33–12.40)	0.447	4.04 (0.61–26.93)	0.149

## Discussion

Our study identifies extensive use of plastic packaging of foods/beverages in slum neighbourhoods of both cities, but particularly Greater Accra (Table 1). This is of concern given low coverage of domestic solid waste management services, particularly in Kisumu^20^. Even where households have waste management services, plastic waste requires appropriate management by municipal or private services. It also provides quantitative evidence that plastic packaging behaviours at retail outlets—and thereby the associated waste generated in the home—are affected not only by the product composition of a typical basket-of-goods, but also by retailer behaviours and local socio-economic and policy context. We find that retail outlet type remained significant even after controlling for food/beverage commodity type, with smaller retail businesses using less plastic packaging in both Greater Accra and Kisumu (Tables 3 and 4). This reflects differing behaviours across retail businesses, with for example informal small businesses (e.g. open markets, kiosks) repackaging bulk products into smaller ‘kadogo’ quantities to increase their affordability for low-income consumers^21^, likely using less plastic when repackaging or reselling. Such retailer behaviour could somewhat mitigate waste mismanagement impacts of low waste collection service coverage among low-income households. However, in doing so, it shifts waste generation from the household to point-of-sale as retailers dispose of packaging when breaking up wholesale purchases or food/beverage multi-packs for sale.

Our study also highlights how local socio-economic, cultural and national policy context affects packaging behaviours. In our study, plastic packaging by vendors or manufacturers was significantly lower in Kisumu than Greater Accra, whilst grocery packaging by consumers was greater in Kisumu (Table 1). In Africa, 46% of countries had legislated to ban plastic bags by 2019^22^, including Kenya, but with a partial ban in Ghana never implemented. The national policy context in Kenya could plausibly have reduced plastic packaging use by retailers, though there are also many other differences between the two case study cities and plastic bag ban enforcement remains challenging among countries such as Kenya^23^. Both countries share common goals concerning plastic waste. Ghana’s 2020 National Plastic Management Policy^24^ seeks to expand EPR, whilst Kenya’s 2022 Sustainable Waste Management Act^25^ legislated for mandatory EPR and domestic waste separation, both yet to be implemented at county level. If the UN 2022 Plastic Treaty^26^ is ratified in 2025, this would form an opportunity for African countries to collaborate and share differing experiences to address plastic pollution effectively.

In our study, AIC goodness-of-fit statistics for logistic regression models based on the NOVA versus country-specific, resident-derived classifications were similar. Thus, our study is inconclusive as to which of these classification approaches is best suited to identifying households lacking waste collection facilities but generating plastic waste from foods via national expenditure surveys. As hypothesised, NOVA group 1 (unprocessed or minimally processed food) and the resident-derived ‘mostly sold in plastics’ class are associated with increased odds of plastic packaging use. However, given relatively low model goodness-of-fit, such classifications provide only an approximate indication of plastic waste generation arising from consumption of a basket-of-goods.

These findings have methodological implications for international monitoring of mismanaged plastics via the many available national household expenditure surveys. They inform potential future extension of current product-specific analyses^11–13^ to cover all products within a household’s basket-of-goods. Firstly, some household expenditure surveys (e.g. the KIHBS) record the retail outlet where goods were purchased^27^, whereas others do not (e.g. the GLSS7)^28^. Given the effect of retail outlet on plastic packaging propensity, the former set of surveys are thus likely better suited to international mismanaged plastic monitoring. The large differences in plastic packaging prevalence and packaging behaviours between Greater Accra and Kisumu (Table 1) also highlight the need for any future multi-country household expenditure survey analyses to be informed by consultation and collaboration with experts familiar with the local socio-economic and policy context. In Kisumu, we analysed two sets of commodity codes, a coarser set comprising fewer codes harmonised with Ghana and a more granular, detailed set from the KIHBS. Whilst logistic regression models using the two classifications had similar predictive performance, packaging characterisation via food codes is subject to the well-known aggregation effect phenomenon^29^. For example, a particular cooking oil sub-type may be sold unpackaged or in reused glass bottles, but the generic cooking oil product group may generally be sold in plastic.

Our survey protocol did not target sampling effort at specific products during market surveillance. Future field studies could instead focus sampling effort on specific products that are widely purchased and of waste management concern, for example short-listing such products via initial stakeholder consultation or reference to national waste hot-spotting reports^30^. For example, Kenya’s hot-spotting report identifies dairy packaging and non-beverage bottles as high absolute contributors to mismanaged plastic waste, with a high proportion of disposable diapers mismanaged^30^. Future studies could also strengthen food/beverage packaging classification through repeated, longer-term consultation with a local expert panel with differing backgrounds (e.g. retailers from open markets, kiosks, and supermarkets), as opposed to the one-off consultation with long-term residents implemented in our study. Since consumption of food-away-from-home is growing in many LMICs^31^, future studies could also examine packaging from food takeaways, as opposed to foods for consumption in the home. To minimise pandemic-related infection risk, our packaging classification evaluation was based on market surveillance at point-of-sale. However, future evaluation studies could administer a household expenditure questionnaire (e.g. from KIHBS or GLSS7 modules) and directly measure waste composition in the home. This would enable direct comparison of the packaging profile of a household’s basket-of-goods with domestic waste composition. Furthermore, regional or national assessments of mismanaged waste composition and hot-spots derived from household surveys could be evaluated through comparison with urban or beach litter surveys, since these have been implemented in many LMICs^32,33^. Particularly where policies to reduce plastic waste generation have been implemented regionally rather than nationwide (e.g. as with Nepal’s carrier bag ban^34^), household surveys could also be used to evaluate such regional policies.

Our study is subject to several other limitations. We did not record or consider consumer characteristics in analysis, nor did we consider plastic packaging by the consumer. The presence of observers could have affected vendor and consumer behaviours, particularly by reducing plastic bag use in Kisumu where bags are banned^35^. Given food composition variation between brands and coarse granularity of commodity coding schemes used in household expenditure surveys, our use of the NOVA classification is subject to ambiguity, a known issue affecting its application^36^. We examined use of plastic packaging, without differentiating by properties such as polymer or colour, which affect recycling and reuse rates^37^. Our analysis also does not take into account packaging efficiency, the amount of plastic used to package a given volume or weight of food or beverage^38^. Packaging efficiency is likely to be greater in lower and middle income than high income countries. For example, multinational companies now explicitly target these markets with sachets as a more lightweight, affordable packaging alternative to PET bottles^14^, whilst budget beverage brands use less PET in bottles than premium brands^38^. Survey errors, such as protocol deviations or mis-recorded observations, could also have reduced estimated performance of NOVA and commodity classification by residents in predicting plastic packaging.

Finally, our cross-sectional study design only captured market transactions in one season, thereby omitting packaging for seasonally available foods. Market surveillance recorded purchases of a subset of commodity codes used by the GLSS7 and KIHBS, not all commodities, so are not nationally representative. This likely reflects regional and seasonal variations in commodity availability and the study’s focus on slums. Similarly, fieldwork took place in the latter stages of the Covid-19 pandemic in late 2021, immediately following non-pharmaceutical intervention measures such as curfews and restrictions on street-hawking in Kenya^39^. Evidence from high income countries such as the UK^40^ suggests these lockdown measures, coupled with consumer concern over infection risk, could have subsequently affected food quantities purchased, product mix, and consumer retail outlet choices. Differences in daily and weekly market surveillance schedules between cities could also have affected comparisons between Accra and Kisumu. However, the effect of food expenditure variability linked to monthly pay-days should be low. This is both because many households in low-income areas pursue informal livelihoods and because fieldwork implementation in both cities extended over more than two months, thereby capturing pay-days.

## Conclusion

Plastic packaging of foods/beverages in low-income neighbourhoods of Greater Accra was far more widespread than in Kisumu. The cities’ contrasting socio-economic contexts, retail and policy environments account for these differences in plastic packaging prevalence. However, whilst Kenya has implemented a ban on selected single-use plastics whilst Ghana has not, lower plastic packaging use in Kisumu cannot be attributed to these policies alone with any uncertainty, given our cross-sectional study design and the many other differences between the case study cities.

Our study shows that plastic packaging use in two low-income urban African cities is approximated by two packaging classifications of commodity codes, but mediated by retailer behaviours and local socio-economic and policy context. We find that packaging classification by local residents and the NOVA classification of unprocessed versus processed foods both predict plastic packaging use with similar, moderate accuracy. There is thus potential via either approach to characterise the packaging profile of an entire household basket-of-goods at national or international level using some of the many available household expenditure survey data sets. However, any future multi-country monitoring study would provide only an approximate indication of plastic waste generation. Such studies should incorporate expert knowledge of national context and the retail environment to reflect the realities of packaging use.

## Methods

### Case study cities

Fieldwork took place in the cities of Greater Accra and Kisumu in Ghana and Kenya, respectively, These case study cities provide contrasting retail environments^41^, given that Accra is a more populous capital city, Kenya’s supermarket sector has expanded very rapidly^42^, and contrasting national policies regulating domestic plastic waste generation. Ghana raises excise duty on semi-finished and raw plastics but has not implemented a single-use plastics ban^43^. Most urban Ghanaian households reported sachet water (sold in plastic bags) as their main drinking-water source in 2022^44^, reflecting government tolerance of single-use plastics. Since 2007, Ghana’s sachet manufacturers have been particularly active in a voluntary Extended Producer Responsibility (EPR) scheme, the Plastic Waste Management Project^45^. In contrast, Kenya fully banned single-use plastic carrier bags in 2017, though with regulatory enforcement challenges^35^. In 2020, Kenya also banned several other single-use plastics, including bottled water, in protected areas^46^. As Kenya’s third largest city, Kisumu has a population of over 500,000. Over 60% of its population lives in informal settlements, typically densely populated and lacking adequate access to electricity, water and sanitation services^47^. In 2022, its population generated an estimated 0.48kg/person/day of household solid waste, mainly organic waste^48^. Urban Greater Accra region’s population was 5.0 million in 2021^49^, with 51% of its households having solid waste collected in 2010^50^. Slum mapping identified 78 slum communities within the city in 2000, though their distribution has subsequently changed^51^. The city of Accra, within Greater Accra region, generates an estimated 0.74kg/person/day of domestic solid waste in 2015 or 1552 tonnes/day in total^52^.

### Sample design for market surveillance survey

**Fig. 1:**
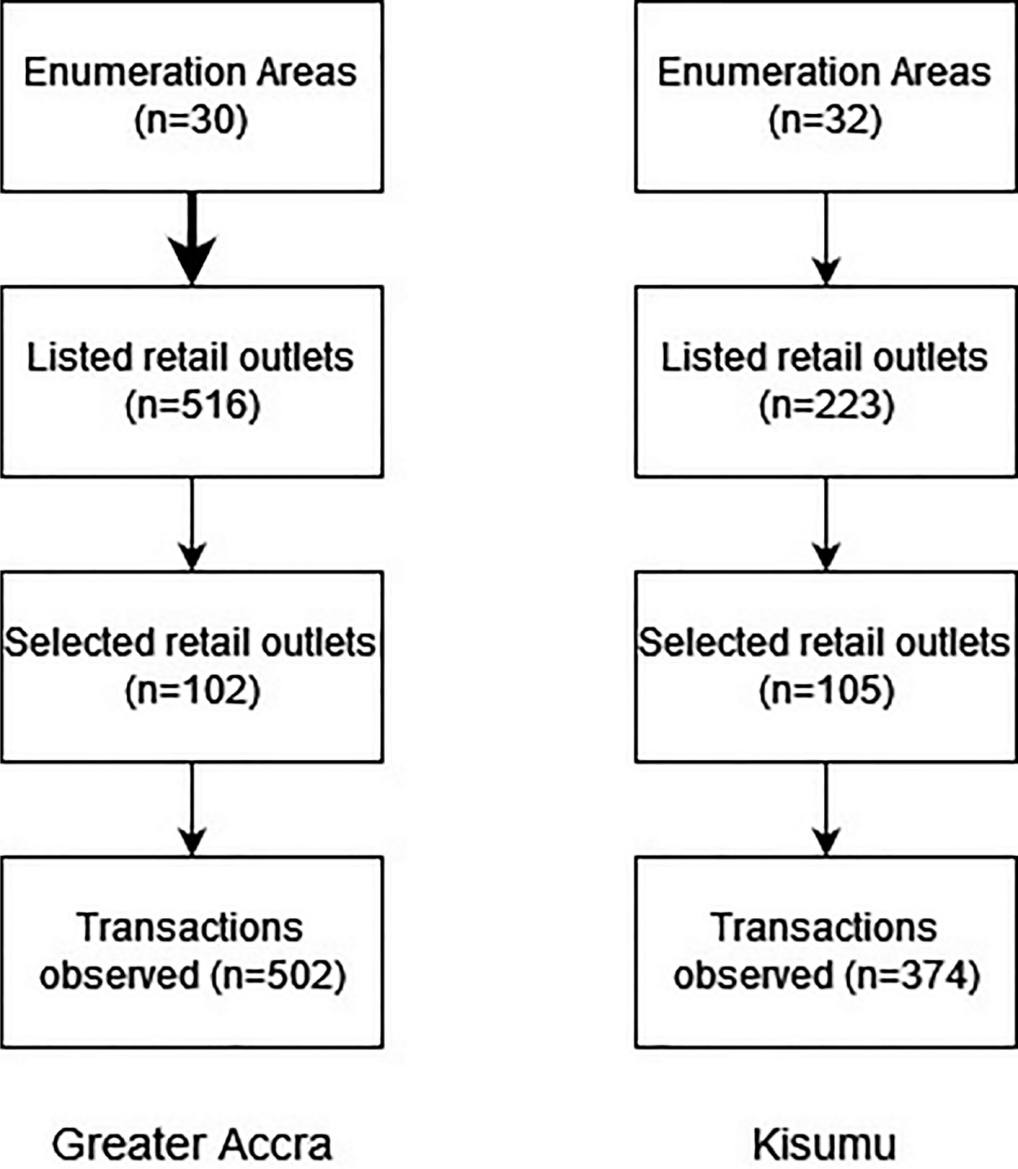
Flow chart, showing the sampling of Enumeration Areas, retail outlets, and observed food or beverage transactions in Kisumu and Greater Accra.

30 and 32 Enumeration Areas (EAs) were randomly selected in each city using probability-proportional-to-size sampling of 2010 and 2009 EAs for Greater Accra region and Kisumu County, respectively (Fig. 1). This EA sample size was chosen to reflect the number of urban EAs selected in Greater Accra region and the former Nyanza province in the 2017 GLSS7^28^ and 2015–16 KIHBS^27^ respectively, both statistically powered to characterise regional consumption. Eligible EAs constituted those classified as urban by national statistical agencies. Within Greater Accra, they also met one or more of the UN-Habitat criteria defining a slum^53^ or lacked solid waste services. Thus, most households in eligible EAs lived in over-crowded or non-durable housing, lacked improved sanitation or water sources, secure tenure, or solid waste management services. EAs dominated by communal establishments were excluded. No equivalent small area statistics were available in Kisumu, so all urban EAs were eligible for initial selection. To allow for replacements, 50 EAs in Kisumu and 70 in Greater Accra were initially selected from lists of eligible EAs. Given that EA boundaries were delineated a decade or more before fieldwork and small area statistics were unavailable in Kenya, survey teams undertook reconnaissance of selected EAs to identify those that no longer met inclusion criteria. Following field reconnaissance, six EAs in Kisumu and 35 EAs in Greater Accra were excluded as lacking slum characteristics and random replacements selected. Two further EAs in Greater Accra were replaced because of concerns for field team security during preliminary fieldwork. Two replacement EAs were also used for pre-testing the survey methodology in each city.

All retail outlets selling foods or beverages in the selected EAs were listed, and then one large (i.e. a supermarket or open market, where present) and one small retail outlet per EA was randomly sampled following listing. Sampling was thus stratified by outlet type. Restaurants, hotels and takeaways were excluded from selection, since food-away-from-home accounts for under 25% of household food expenditure in both cities^54,55^. Following initial retail outlet sampling, it became apparent that larger retail outlets (i.e. open markets, supermarkets) were under-represented. Therefore, a follow-up exercise additionally listed and sampled open markets and supermarkets nearest to the selected EAs. In Greater Accra, three supermarkets and 22 open air markets were sampled via this follow-up exercise, with eight supermarkets and 42 open air markets sampled in Kisumu. In Kisumu, a total of 223 eligible retail outlets were listed, with 104 randomly selected for data collection. 508 retail outlets were listed in Greater Accra, of which 102 were visited for the market survey.

### Market surveillance survey

Via a cross-sectional survey, field surveyors observed how shoppers purchased food and beverage products from the selected retail outlets. The surveyors first explained the project to retail outlet staff, seeking their informed consent to make observations and purchase samples of foods/beverages sold. Surveyors observed how foods and beverages were packaged, approximate quantities purchased, and commodity type for transactions by the first five customers to visit each outlet on one occasion. Observations, lasting approximately an hour at each retail outlet, recorded transactions during a median proportion of 10.2% of opening hours for Kisumu’s shops, supermarkets and kiosks and 5.8% for its open markets. In Greater Accra, market surveillance observations were made over a median of approximately 2 h per retail outlet. Most observations (84.7%) occurred from 8 am to 1 pm and on weekdays in Kisumu and from 1 pm to 5 pm in Greater Accra (84.3%) on Mondays to Thursdays. The different timings in Greater Accra resulted from field logistical difficulties in travelling across Accra as a larger, more congested city. Observation timings also reflected busier retail periods in each city. Surveyors recorded whether items were pre-packaged or packaged by the vendor and type of packaging material. Following transaction observations, surveyors purchased samples of products sold in plastic packaging for subsequent desk-based characterisation. This included assessment of whether plastic packaging had been reused, independently recorded by five observers in Kisumu and four observers in Greater Accra to enable assessment of inter-observer agreement.

To ensure consistency in market survey implementation, field teams were first trained in market surveillance implementation over two days, with protocols piloted in an EA not selected for the main study. Spot-checks were undertaken by project investigators and supervisors, who visited randomly selected stores during fieldwork. Use of SurveyCTO for computer-assisted data capture additionally enabled encoding of questionnaire flow logic and range checks into data capture forms, alongside rapid data review following each day’s fieldwork.

Commodity classifications in market surveillance mirrored the most recent national household expenditure surveys in each country. In Kisumu, commodities observed were classified using 193 food or beverage codes, following coding used in the KIHBS^27^. In initial pre-testing in Greater Accra, the full set of 291 commodity codes from the GLSS7^28^ was used. However, this proved challenging, so a less granular set of 72 food or beverage codes (following commodity groupings in the GLSS7 questionnaire) was used instead. For inter-city comparison, a look-up table was created to harmonise KIHBS commodity codes with the 72 GLSS7 codes for Greater Accra. Retail outlets were classified using a KIHBS typology, adapted for use in Greater Accra where the GLSS7 had no equivalent typology.

All data were collected using hand-held tablets via computer-assisted interviewing using the SurveyCTO software^56^. Fieldwork in Ghana occurred from 31^st^ August to 24^th^ November 2021 and in Kenya from 17th August to 21st October 2021.

The study was approved by the Faculty of Environmental and Life Sciences Ethical Review Committee, University of Southampton, UK (reference: 55755; approval date 19th August 2020), by the Ethics Review Office of Jaramogi Oginga Odinga University of Science and Technology, Kenya (REF: ERC/23/6/20-4; approval date 19th August 2020), and by the Institutional Review Board of the Noguchi Memorial Institute for Medical Research, University of Ghana (Ref: 003/20-21; approval date: 2nd September 2020). Informed consent was sought from participants and the study was conducted in accordance with the Vancouver guidelines and Helsinki Declaration.

### Food and beverage classification

Detailed foods and beverage codes were reclassified using three different classifications. Firstly, the FAO/WHO Global Individual Food consumption data Tool (FAO/WHO GIFT)^57^, a tool for food consumption survey harmonisation, was used to combine commodities observed in Ghana versus Kenya into groups for initial exploratory analysis. Secondly, foods and beverages were grouped using the NOVA classification^18^. As noted above, this differentiates four categories based on degree of processing: unprocessed and minimally processed foods (group 1); processed culinary ingredients (group 2); processed foods (group 3); and ultra-processed foods (group 4). We hypothesised that processing and plastic packaging would be correlated, with NOVA groups 3 and 4 the most frequently packaged in plastics and group 1 the least. The Open Food Facts database^58^, an international database that classifies foods into NOVA groups, was used to assist with assigning NOVA classes to commodities. Next, a local resident familiar with each city’s retail environment classified each food into three groups: mostly packaged by the retailer or manufacturer in plastic, sometimes packaged in plastic, or never packaged in plastic. Both residents were male professionals and long-term residents of each city with personal familiarity of low-income, working in the telecommunications and health sector in Greater Accra and Kisumu respectively. Neither resident had any knowledge of the market surveillance survey. In Kenya, the NOVA, GIFT and plastic packaging classifications were applied both to the more detailed food/beverage codes from the KIHBS and to the less detailed, harmonised codes.

### Statistical analysis

Differences in transaction characteristics for Greater Accra versus Kisumu were compared using chi square tests. The two in-country residents’ classifications of harmonised commodity codes were cross-tabulated, with the Kappa index calculated as a measure of agreement^19^. Resident-derived and NOVA classifications were also compared via Fisher’s exact test given small cell counts. Significance levels (α = 0.05) were adjusted to account for multiple testing using the Bonferroni correction, being α = 0.006 for inter-city comparison of transaction characteristics, α = 0.007 for kappa statistics, and α = 0.013 for Fisher’s exact test.

In Kisumu and Greater Accra, follow-up, desk-based characterisation by the survey team identified evidence of packaging reuse among only 3 (1.7%) of 180 plastic samples collected in Greater Accra and 17 (4.9%) of 347 samples collected in Kisumu. African plastic packaging is typically inconsistently labelled with recycling information, given lack of harmonised labelling guidelines and limited regulatory capacity^59^. Therefore, the desk-based observation protocol did not record whether packaging samples were labelled as being recycled plastics. Thus, subsequent analysis considered use of any plastic packaging by manufacturers or vendors, as opposed to single-use plastic packaging (which would necessitate exclusion of reused or recycled plastics).

In Kenya, logistic regression modelling was used to examine associations between plastic packaging use and resident-derived and NOVA commodity classifications. Separate models predicted plastic packaging by either retailers or commodity manufacturers versus manufactured pre-packaging only. The resident-derived classification of coarse, harmonised commodity codes was the only classification available for analysis in Greater Accra, but in Kisumu, we analysed both the resident-derived classification of harmonised codes and that based on the more detailed KIHBS codes. Robust logistic regression models, accounting for clustering in transaction observations by retail outlet, were used to assess the relationship between plastic packaging use and NOVA food groups and the resident-derived packaging classes for each city. Following initial univariate model fitting, multivariate models of commodity classifications then adjusted for retail outlet type and quantity of commodity purchased. The former thus reflected retailer packaging behaviours, whilst the latter reflected Africa’s ‘kadogo’ economy (meaning small in Kiswahili). The ‘kadogo’ economy is characterised by vendors repackaging and re-pricing basic essentials like food into small sizes consistent with slum-dwellers’ paying capacity and consumption-smoothing^21^. Kadogo transactions were identified as those in the smallest quartile for a given commodity and measurement unit (pieces, weight or volume). Supermarket and shop categories were grouped for Kenyan modelling, given both had similar packaging characteristics. NOVA and resident-derived commodity classes were collapsed where data were sparse. Adjusted models incorporated all terms initially, retaining those significant at alpha < 0.1 and was informed by goodness-of-fit diagnostics, such as chi square statistics for covariate patterns^60^. Goodness-of-fit for models based on residents’ versus NOVA classifications was compared using the Akaike Information Criterion (AIC), following standard logistic regression model-fitting practice^61^. Analyses were undertaken in Stata v18^62^.

## Supplementary Information

Supplementary Tables.

## Data Availability

Wanza, P., Amponsah, M., Damkjaer, S., Amoah, J., Boafor, E., Dzodzomenyo, M., Myers-Hansen, G.A, Oigo, J., Okotto, L., Shaw, P., Umar, F., Wright, J., Okotto-Okotto, J. (2024). Market Survey of Food and Beverage Purchase Behaviours, Commodity Packaging and Plastics in Off-Grid Greater Accra, Ghana and Kisumu, Kenya, 2021. [Data Collection]. Colchester, Essex: UK Data Service 10.5255/UKDA-SN-856834.
