# Supplementary material for: Food classifications provide an approximate packaging indicator to support monitoring of mismanaged plastic waste
Source: Sci Rep. 2025 Feb 11;15:5041. doi: 10.1038/s41598-025-89350-0 (PMC11814282; doi:10.1038/s41598-025-89350-0)
Supplement: Supplementary file 1 — Supplementary Tables. [file 41598_2025_89350_MOESM1_ESM.docx]

# Food classifications provide an approximate packaging indicator to support monitoring of mismanaged plastic waste.

Mawuli Dzodzomenyo^1^, Moses Asamoah^1, 2^, Joseph Okotto-Okotto^3^, Lorna-Grace Okotto^4^, Peggy Wanza^3^, Gustavus A. Myers-Hansen^1^, Jim Wright^5*^

^1^ School of Public Health, University of Ghana, Accra, Ghana.

^2^ Social Statistics and Demography, University of Southampton, Southampton, UK.

^3^ Victoria Institute for Research on Environment and Development International, Rabuor, Kenya.

^4^ School of Spatial Planning and Natural Resource Management, Jaramogi Oginga Odinga University of Science and Technology, Bondo, Kenya.

^5^ School of Geography and Environmental Science, University of Southampton, Southampton, UK.

^*^ Corresponding author. Email: j.a.wright@soton.ac.uk

|  | **Unadjusted** | | **Adjusted** | |
| --- | --- | --- | --- | --- |
| **Transaction characteristics** | **Odds ratio (95% CI)** | **P value** | **Odds ratio (95% CI)** | **P value** |
| Small (‘kadogo’) transactions |  |  |  |  |
| Quantity purchased below 25^th^ centile for commodity | 0.57 (0.27-1.24) | 0.156 |  |  |
| Facility type (reference: supermarket) |  |  |  |  |
| Open market | 0.94 (0.61-1.45) | 0.786 |  |  |
| Kiosk | 0.13 (0.04-0.44) | 0.001 |  |  |
| NOVA food group (reference: Group 1 unprocessed foods) |  |  |  |  |
| Group 2: Processed culinary ingredients | 2.33 (1.42-3.82) | 0.001 | 1.95 (1.18-3.24) | 0.009 |
| Group 3 or 4: processed or ultra-processed foods | 5.56 (3.21-9.64) | <0.001 | 4.87 (2.78-8.52) | <0.001 |
| Local expert classification: harmonised commodity codes (reference: mostly sold in plastics) |  |  |  |  |
| Sometimes sold in plastics | 0.56 (0.30-1.06) | 0.075 | 0.51 (0.27-0.94) | 0.032 |
| Seldom sold in plastics | 0.04 (0.01-0.25) | 0.001 | 0.03 (0.00-0.19) | <0.001 |
| Local expert classification: KIHBS commodity codes (reference: mostly sold in plastics)  Sometimes/seldom sold in plastics | 2.10 (1.33-3.33) | 0.002 | 2.49 (1.60-3.86) | <0.001 |

**Supplementary Table S1:** Odds ratios from unadjusted and adjusted logistic regression models, predicting plastic packaging of foods and beverages by manufacturers in Kisumu, excluding plastic packaging use by retailers.

| **City** | **Type of packaging predicted** | **NOVAN** | **Harmonised expert commodity classification** | **Detailed (KIHBS) expert commodity classification** |
| --- | --- | --- | --- | --- |
| Kisumu | Plastic packaging by retailer or manufacturer | 1.289 | 1.245 | 1.284 |
| Kisumu | Plastic packaging by manufacturer only | 1.241 | 1.248 | 1.289 |
| Greater Accra | Plastic packaging by retailer or manufacturer | 0.421 | 0.443 | - |
| Greater Accra | Plastic packaging by manufacturer only | 0.569 | 0.577 | - |

**Supplementary Table S2.** Aikaike Information Criteria goodness-of-fit statistics for adjusted logistic regression models predicting plastic packaging of foods and beverages based on three different commodity classifications.

|  | **Unadjusted** | | **Adjusted** | |
| --- | --- | --- | --- | --- |
| **Transaction characteristics** | **Odds ratio** | **P value** | **Odds ratio** | **P value** |
| Small (‘kadogo’) transactions |  |  |  |  |
| Quantity purchased below 25^th^ centile for commodity | 1.47 (0.46-4.65) | 0.516 |  |  |
| Facility type (reference: supermarket) |  |  |  |  |
| Open market | 0.25 (0.08-0.77) | 0.016 |  |  |
| Shop | 0.30 (0.11-0.84) | 0.022 |  |  |
| NOVA food group (reference: Group 1 unprocessed foods) |  |  |  |  |
| Group 2: Processed culinary ingredients | 0.83 (0.36-1.91) | 0.663 | 0.85 (0.36-2.05) | 0.723 |
| Group 3 or 4: processed or ultra-processed foods | 2.68 (1.17-6.11) | 0.020 | 2.59 (1.13-5.91) | 0.024 |
| Local expert classification: (reference: mostly sold in plastics) |  |  |  |  |
| Sometimes sold in plastics | 0.35 (0.17-0.74) | 0.006 | 0.52 (0.25-1.09) | 0.084 |
| Seldom sold in plastics | 0.59 (0.22-1.55) | 0.281 | 0.57 (0.28-3.11) | 0.913 |

**Supplementary Table S3.** Odds ratios from unadjusted and adjusted logistic regression models, predicting plastic pre-packaging of foods and beverages observed at retail outlets in Greater Accra
